# Supplementary material for: Single-cell and bulk RNA sequencing analysis reveals CENPA as a potential biomarker and therapeutic target in cancers
Source: PLoS One. 2025 Jan 16;20(1):e0314745. doi: 10.1371/journal.pone.0314745 (PMC11737691; doi:10.1371/journal.pone.0314745)
Supplement: S2 Table — (DOCX) [file pone.0314745.s002.docx]

**S-Table 2. Cox regression analysis of CENPA and clinical characteristics in glioma**

| **Characteristics** | **Total(N)** | **Univariate analysis** | |  | **Multivariate analysis** | |
| --- | --- | --- | --- | --- | --- | --- |
|  |  | **Hazard ratio (95% CI)** | **P value** |  | **Hazard ratio (95% CI)** | **P value** |
| CENPA | 527 | 1.582 (1.412-1.772) | **<0.001** |  | 1.244 (1.071-1.446) | **0.004** |
| IDH status | 524 |  |  |  |  |  |
| WT | 97 | Reference |  |  |  |  |
| Mut | 427 | 0.186 (0.130-0.265) | **<0.001** |  | 0.386 (0.242-0.616) | **<0.001** |
| 1p/19q codeletion | 527 |  |  |  |  |  |
| non-codel | 357 | Reference |  |  |  |  |
| codel | 170 | 0.401 (0.256-0.629) | **<0.001** |  | 0.929 (0.511-1.691) | 0.810 |
| Primary therapy outcome | 457 |  |  |  |  |  |
| PD | 110 | Reference |  |  |  |  |
| SD | 146 | 0.439 (0.292-0.661) | **<0.001** |  | 0.511 (0.329-0.793) | **0.003** |
| PR | 64 | 0.175 (0.076-0.402) | **<0.001** |  | 0.185 (0.078-0.437) | **<0.001** |
| CR | 137 | 0.122 (0.056-0.266) | **<0.001** |  | 0.161 (0.072-0.360) | **<0.001** |
| Gender | 527 |  |  |  |  |  |
| Female | 238 | Reference |  |  |  |  |
| Male | 289 | 1.124 (0.800-1.580) | 0.499 |  |  |  |
| Race | 516 |  |  |  |  |  |
| White | 486 | Reference |  |  |  |  |
| Black or African American | 22 | 1.459 (0.680-3.131) | 0.333 |  |  |  |
| Asian | 8 | 0.000 (0.000-Inf) | 0.993 |  |  |  |
| Age | 527 |  |  |  |  |  |
| <=40 | 264 | Reference |  |  |  |  |
| >40 | 263 | 2.889 (2.009-4.155) | **<0.001** |  | 3.505 (2.296-5.350) | **<0.001** |
| Histological type | 527 |  |  |  |  |  |
| Astrocytoma | 195 | Reference |  |  |  |  |
| Oligoastrocytoma | 134 | 0.661 (0.421-1.037) | 0.071 |  | 1.305 (0.789-2.159) | 0.299 |
| Oligodendroglioma | 198 | 0.577 (0.392-0.848) | **0.005** |  | 0.535 (0.333-0.860) | **0.010** |
| Laterality | 522 |  |  |  |  |  |
| Left | 256 | Reference |  |  |  |  |
| Midline | 6 | 1.007 (0.304-3.337) | 0.991 |  |  |  |
| Right | 260 | 0.771 (0.543-1.094) | 0.145 |  |  |  |

*TCGA (LGG+GBM) cohort were analyzed.
