# Supplementary material for: Single-cell and bulk RNA sequencing analysis reveals CENPA as a potential biomarker and therapeutic target in cancers
Source: PLoS One. 2025 Jan 16;20(1):e0314745. doi: 10.1371/journal.pone.0314745 (PMC11737691; doi:10.1371/journal.pone.0314745)
Supplement: S3 Table — (DOCX) [file pone.0314745.s003.docx]

**S-Table 3. Computational drug predictions of CENPA**

| **Protein** | **Drug** | **Correlation** | **FDR** | **Vina score** |
| --- | --- | --- | --- | --- |
| CENPA | CD-437 | -0.169974692 | 6.5E-06 | -9.9 |
| CENPA | 3-Cl-AHPC | -0.18397462 | 9.9E-07 | -9.1 |
| CENPA | Trametinib | 0.230107851 | 3.6E-11 | -8.8 |
| CENPA | BI-2536 | -0.168477555 | 4.5E-06 | -8.3 |
| CENPA | GSK461364 | -0.170673879 | 5.7E-06 | -8.2 |
| CENPA | KX2-391 | -0.166982975 | 9.8E-06 | -7.8 |
| CENPA | manumycin A | -0.172313159 | 4.4E-06 | -7.6 |
| CENPA | selumetinib | 0.194228307 | 1.7E-08 | -6.6 |
| CENPA | PD-0325901 | 0.168973707 | 6.1E-06 | -6.5 |
| CENPA | RDEA119 | 0.207186818 | 1.4E-09 | -6 |
| CENPA | cerulenin | -0.170490806 | 6.9E-06 | -5 |
| CENPA | triazolothiadiazine | -0.178487698 | 1.2E-06 | -3.7 |
