# Supplementary material for: Single-cell and bulk RNA sequencing analysis reveals CENPA as a potential biomarker and therapeutic target in cancers
Source: PLoS One. 2025 Jan 16;20(1):e0314745. doi: 10.1371/journal.pone.0314745 (PMC11737691; doi:10.1371/journal.pone.0314745)
Supplement: S1 Fig — TCGA cohort was analyzed. (DOCX) [file pone.0314745.s004.docx]

**
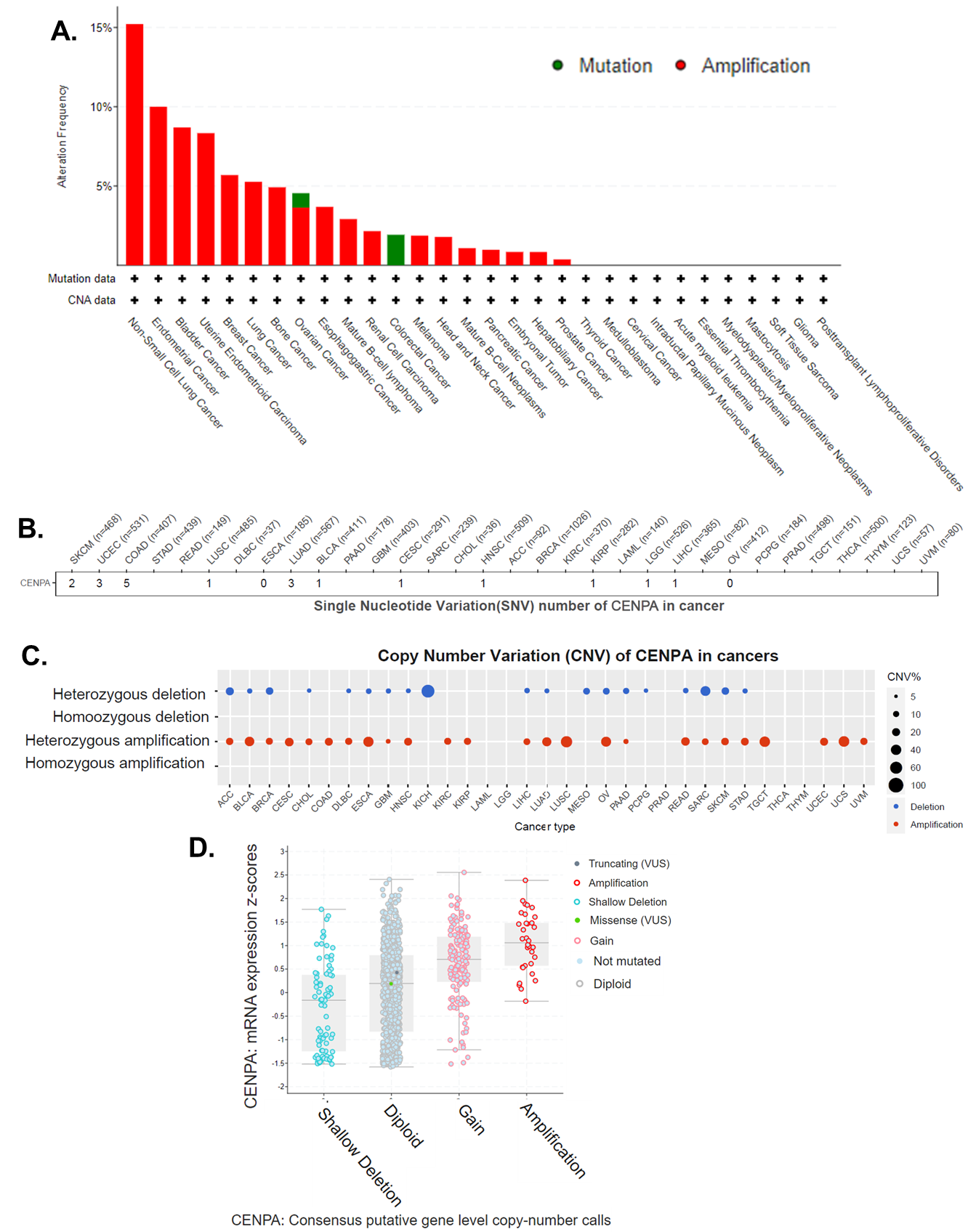
**

**S-Figure 1. Alteration of CENPA in cancers genome. TCGA cohort was analyzed.**

**A.** The CENPA gene alteration frequency of different types of cancers. **B.** Single-nucleotide variant number of CENPA in cancers. Numbers in the box represent the number of samples that have single-nucleotide variant CENPA in corresponding cancers. The “0” indicates that there is no mutation in the CENPA coding region, and the blank indicates that there is no mutation in all regions of the CENPA gene. **C.** Copy number variation percentage plot of CENPA in cancers. **D.** The expression of CENPA in different gene alteration groups.
