# Supplementary material for: Single-cell and bulk RNA sequencing analysis reveals CENPA as a potential biomarker and therapeutic target in cancers
Source: PLoS One. 2025 Jan 16;20(1):e0314745. doi: 10.1371/journal.pone.0314745 (PMC11737691; doi:10.1371/journal.pone.0314745)
Supplement: S2 Fig — (DOCX) [file pone.0314745.s005.docx]

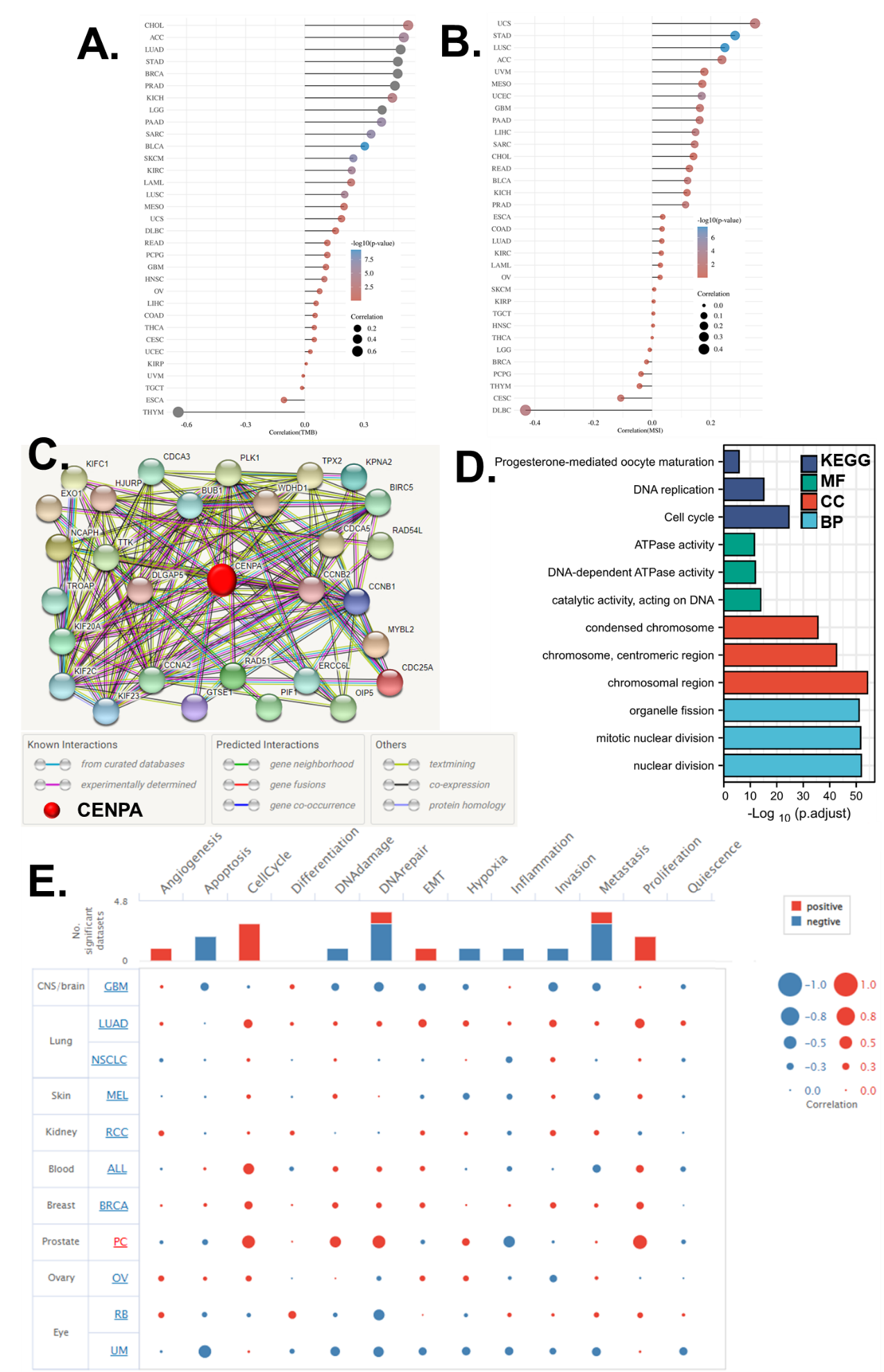


**S-Figure 2. Functional and mutation associations of CENPA in cancers.**

**A.** The correlations of CENPA expression and tumor mutation burden (TMB) in TCGA. **B.** The correlation of CENPA expression and microsatellite instability (MSI) in TCGA. **C.** Protein-protein interaction network of top 30 CENPA-correlated genes. The top 30 CENPA correlated genes were identified using the GEPIA based on all TCGA cancer data. The network was constructed using the STRING. The minimum required interaction score was set at high confidence (>0.9). **D.** GO terminology and KEGG Pathway enrichment analysis of top 200 CENPA-correlated genes. The top 200 CENPA correlated genes were identified using the GEPIA based on all TCGA cancer data. **E.** Correlations of CENPA expression and functional scores in single-cell data sets. The single-cell data was accessed and analyzed using the CancerSEA.
