# Supplementary material for: Single-cell and bulk RNA sequencing analysis reveals CENPA as a potential biomarker and therapeutic target in cancers
Source: PLoS One. 2025 Jan 16;20(1):e0314745. doi: 10.1371/journal.pone.0314745 (PMC11737691; doi:10.1371/journal.pone.0314745)
Supplement: Attachment — Submitted filename: comments.docx [file pone.0314745.s006.docx]

Reviewer #1: The manuscript presents a comprehensive bioinformatic analysis of CENPA expression across various cancers, evaluating its potential as a biomarker for cancer diagnosis and prognosis. The authors have conducted a thorough investigation, examining not only CENPA's roles in cancer but also its correlation with the tumor microenvironment. Additionally, they have performed screening to identify potential drugs targeting CENPA in cancer cells.

The study design is logical and well-structured, with comprehensive and convincing data analysis that supports the conclusions drawn.

To further enhance the manuscript, I suggest the following revisions:

1. In sections 2, 7, and 9, please write out the full names of cancer types rather than using abbreviations. This would improve readability for a broader audience.

We have written out the full names of cancer types rather than just using abbreviations.

2. The cell type labels in Figure 2A are currently too small to read clearly. Please increase the font size to ensure legibility.

We have increased the size of the cell type labels.

3. The resolution of Figure 5B needs improvement, as the current image appears slightly blurry. A higher-resolution version would better showcase the results.

We have increased the resolution of Figure 5B.

Overall, these minor revisions would enhance the presentation of this otherwise well-executed study.

Reviewer #2: Here, a pan-cancer bioinformatic analysis has been performed on centromere protein A (CENPA). The manuscript analyzed different aspects in different cancer types without a logical procedure.

We appreciate your comment and understand your concern about the logical procedure. Therefore, we have rearranged the order of presenting the results, now the paper follows logic below:

1 comparison between cancer non-cancer to support the diagnostic value

2 analysis of survival association for supporting the prognostic value

3 glioma as a representative cancer typo to support the practical clinical application of CENPA for prognosis.

4 explore the mechanism underlying the impact of CENPA on cancer,

1) first, we find it is expressed in malignant cells not immune cells
2) it is expressed in nucleur
3) it is highly associated with cancer cell stemness (proliferation)

5 explore the how the gene impact the immune cells and exam if it is useful for prediction of immune therapy.

6 drug prediction of CENPA in cancers

The methods used are not well described,

We have modified the method description and included much more details. If it is still need to be improve, please specify and we are happy to modify it.

the results are confusing and do not support the conclusions.

We have rearranged the results and modified the conclusion to

“CENPA holds promise as a biomarker in cancers linked to cell cycle regulation and stemness, with significant potential for diagnostic, prognostic, and therapeutic applications.”

We also modified the title and included “potential” to avoid overstate of the conclusion.
